# Staphylococcal Bacterial Persister Cells, Biofilms, and Intracellular Infection Are Disrupted by JD1, a Membrane-Damaging Small Molecule

**DOI:** 10.1128/mBio.01801-21

**Published:** 2021-10-12

**Authors:** Jamie L. Dombach, Joaquin L. J. Quintana, Corrella S. Detweiler

**Affiliations:** a Department of Molecular, Cellular, and Developmental Biology, University of Colorado Boulder, Boulder, Colorado, USA; University of Michigan—Ann Arbor

**Keywords:** Gram-positive bacteria, membrane structure, *Staphylococcus*, biofilms, persister cells, intracellular infection, *Salmonella*, antibacterial, bactericidal activity, cell membranes, Gram-negative bacteria, intracellular pathogen, membrane potential, outer membrane

## Abstract

Rates of antibiotic and multidrug resistance are rapidly rising, leaving fewer options for successful treatment of bacterial infections. In addition to acquiring genetic resistance, many pathogens form persister cells, form biofilms, and/or cause intracellular infections that enable bacteria to withstand antibiotic treatment and serve as a source of recurring infections. JD1 is a small molecule previously shown to kill Gram-negative bacteria under conditions where the outer membrane and/or efflux pumps are disrupted. We show here that JD1 rapidly disrupts membrane potential and kills Gram-positive bacteria. Further investigation revealed that treatment with JD1 disrupts membrane barrier function and causes aberrant membranous structures to form. Additionally, exposure to JD1 reduced the number of Staphylococcus aureus and Staphylococcus epidermidis viable persister cells within broth culture by up to 1,000-fold and reduced the matrix and cell volume of biofilms that had been established for 24 h. Finally, we show that JD1 reduced the number of recoverable methicillin-resistant S. aureus organisms from infected cells. These observations indicate that JD1 inhibits staphylococcal cells in difficult-to-treat growth stages as well as, or better than, current clinical antibiotics. Thus, JD1 shows the importance of testing compounds under conditions that are relevant to infection, demonstrates the utility that membrane-targeting compounds have against multidrug-resistant bacteria, and indicates that small molecules that target bacterial cell membranes may serve as potent broad-spectrum antibacterials.

## INTRODUCTION

Few new antibiotics with novel structures or functions have been approved for use over the last several decades, limiting the treatment options for bacterial infections (1). In addition, since many existing antibiotics bind the same target in similar locations, resistance to one antibiotic quickly enables cross-resistance to multiple antibiotics, notably for macrolides, lincosamides, and streptogramins and for β-lactams (2). Due to the rapid development of resistance, the few antibiotics that are effective against multidrug-resistant (MDR) bacteria are reserved for use in critical cases. These antibiotics are referred to as last line of defense or last resort antibiotics and often have severe side effects that limit their utility (3, 4).

The increase in antibiotic resistance is occurring in both Gram-positive and Gram-negative bacteria, which use common cellular mechanisms, growth stages, and infection strategies to evade antibiotics and host immunity. For instance, toxic molecules, including antibiotics, are exported by efflux pumps. However, Gram-negative bacteria are especially difficult to kill due to their outer membrane, which blocks many compounds from entering the cell (5).These bacteria primarily use efflux pumps of the resistance-nodulation-division (RND) family to export antibiotics (6, 7). In addition to expelling antibiotics, efflux pumps have an important role during infection, as they export host-derived molecules that would otherwise damage the bacterial cell, including the Gram-negative outer membrane (8, 9).

Shared mechanisms of host evasion include the establishment of a persister state, biofilms, and an intracellular lifestyle. Bacterial persister cells may be defined as nongrowing and/or metabolically less active cells that constitute a small population in all cultures of bacteria but can increase in proportion (10). For example, 100% of Staphylococcus cultures become persister cells with growth at high density. When environmental conditions become favorable, persister cells exit their metabolically quiescent state and resume active growth (11–14). Their decreased metabolism protects persister cells from most currently available antibiotics, which interfere with processes that only occur, or occur more rapidly, in metabolically active or actively growing cells (15). Due to their intrinsically high resistance to antibiotics, persisters are an important cause of recurring infections (15). Persister cells also reside within biofilms and may reside intracellularly during infection. Biofilms are notoriously difficult to treat due to an extracellular matrix that excludes many compounds, including antibiotics (12, 16). The biofilms of Staphylococcus epidermidis are particularly robust and grow on medical implants (16, 17). In contrast, Staphylococcus aureus resides both extracellularly and intracellularly during infection (18, 19). Intracellular pathogens evade neutralizing components of the host immune system such as antibodies and neutrophils, allowing them refuge in a protected environment (20, 21). Additionally, intracellular pathogens may cause recurring infections by escaping antibiotic treatment, as concentrations of antibiotics tend to be lower inside host cells than in the extracellular environment (22).

Compounds with activity against the bacterial cell membrane may be effective for treating cells in a persister state, in biofilms, and within host cells, because at all times the cell membrane is essential (23–26). The lipid composition of bacterial cell membranes is distinct from that of mammalian cell membranes and is mainly composed of phosphatidylethanolamine (PE), phosphatidylglycerol, and cardiolipin, although there are differences in membrane composition across bacteria. For instance, glucolipids are a feature of Gram-positive bacteria (27), and S. aureus has very little PE (28). Nevertheless, bacterial cell membranes have a more negative overall charge than mammalian cell membranes, which are mainly composed of phosphatidylcholine, PE, phosphatidylserine, and cholesterol (25, 29). The outer leaflet of mammalian cell membranes has a more overall neutral charge, in part because negatively charged lipids tend to be within the inner leaflet (25). These distinctions indicate that small molecules that preferentially target bacterial membranes over mammalian membranes may exist.

JD1 (406 g/mol) is a small aromatic molecule that facilitates the killing of the Gram-negative pathogen Salmonella enterica serovar Typhimurium in mammalian macrophages and epithelial cells and reduces bacterial load in mice. In broth culture, JD1 damages the inner membrane if conditions permeabilize the outer membrane and/or the RND family AcrAB-TolC efflux pump is inactivated (30). We therefore examined the effect of JD1 on Gram-positive bacteria, which lack an outer membrane and may facilitate JD1 access to the cell membrane in persister cells and biofilms. We began with traditional laboratory model organisms (Bacillus subtilis and S. aureus Newman) and progressed to more virulent strains. Staphylococcus species tested included S. aureus and S. epidermidis, both of which form persister cells and biofilms, but only S. aureus causes intracellular infections. S. aureus FDA209 was isolated from a skin lesion, is sensitive to antibiotics, and has historically been used in antimicrobial and quality control testing (31–33). S. aureus HG001 is a common lab strain derivative of NCTC835, a methicillin-sensitive strain isolated from a septic patient but with a restored *rsbU^+^* gene to increase its biofilm production (34). S. aureus USA300 grows rapidly and is a community-acquired methicillin-resistant strain that causes intracellular infections (35). Finally, we examined S. epidermidis 1457, which is antibiotic sensitive and forms robust biofilms (36).

Here, we show that JD1 disrupts the staphylococcal cell membrane and interrupts growth states that are critical to infections. Through the use of fluorescent probes, we found that JD1 increases the curvature and/or the fluidity of the cellular membrane of S. aureus and leads to a breakdown of barrier function. Using superresolution structured illumination microscopy (SR-SIM) and transmission electron microscopy (TEM), we also found that JD1 treatment results in the formation of membrane distortions. JD1 decreased the number of viable persister cells in broth, reduced established staphylococcal biofilms, and decreased the number of recoverable bacteria from intracellular infections. These data show the importance of testing novel compounds not just in broth culture but also in growth stages germane to infection and suggest that small, membrane-active molecules may serve as potent broad-spectrum antibacterials.

## RESULTS

### JD1 is bacteriostatic and bactericidal against Gram-positive bacteria.

**FIG 1:**
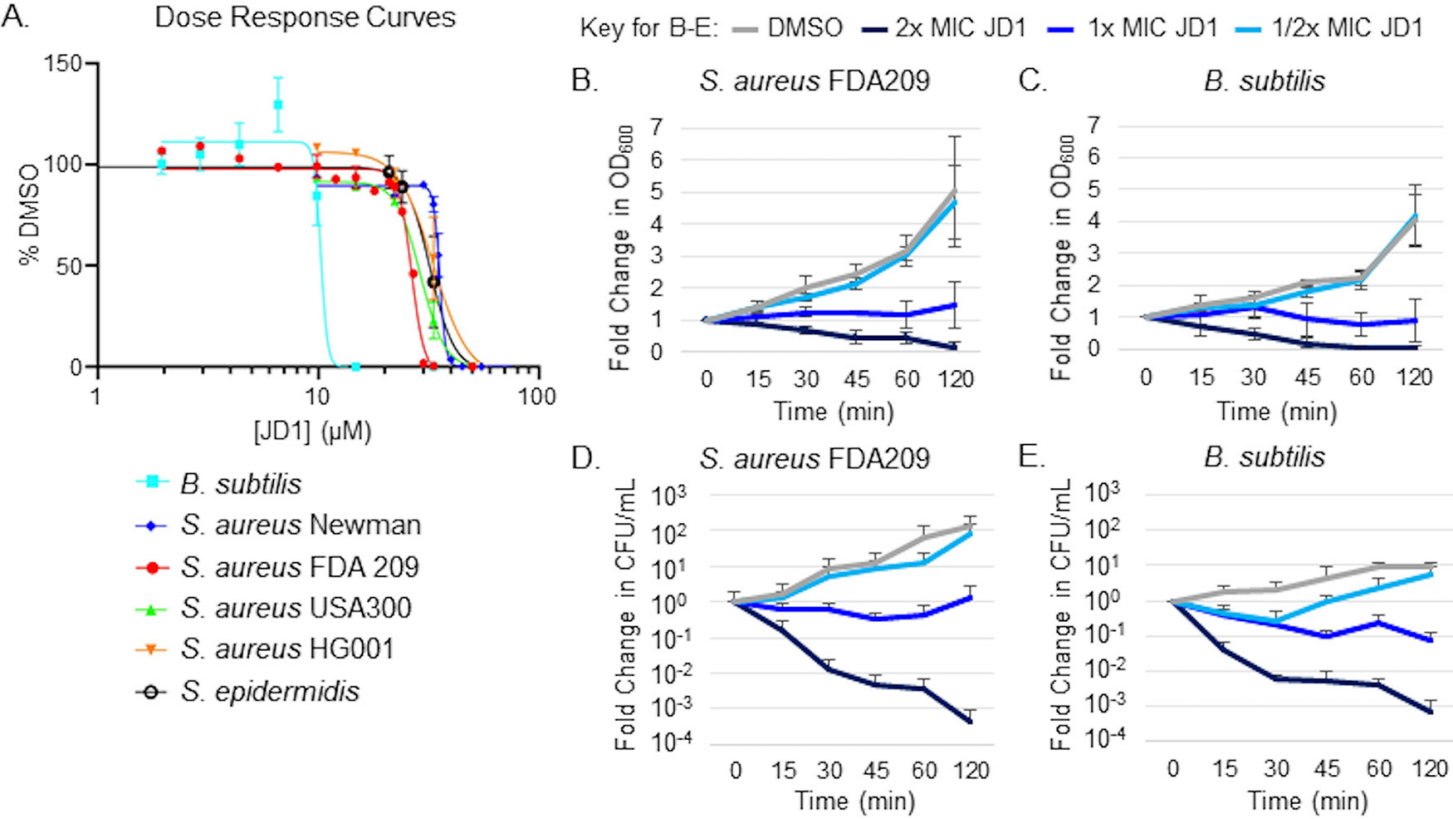
JD1 is bacteriostatic and bactericidal against Gram-positive bacteria. (A) Dose-response curves monitoring bacterial growth of the indicated strains from an OD_600_ of 0.01 in LB, normalized to growth in 2% DMSO. Means and standard errors of the means (SEM) for at least three biological replicates performed with technical triplicates are shown. (B to E) Growth curves and kill curves. Mid-log-phase LB-grown cultures of the indicated strains were treated at time zero with either DMSO or the corresponding MIC_95_ of JD1 (Table 1). Cultures were monitored for OD_600_ (B and C) and plated for enumeration of CFU (D and E). Data are presented as fold change. Means and SEM for three biological replicates performed with technical triplicates are shown.

We established whether JD1 is potent against Gram-positive bacteria in standard broth compared to dimethyl sulfoxide (DMSO) (solvent control). When Bacillus subtilis (ATCC 6633) was grown in a rich medium (LB), JD1 inhibited growth with a MIC_95_ of 12.5 μM. The MIC_95_ is defined as the concentration required to inhibit 95% of bacterial growth after 18 h. Under the same conditions, the MIC_95_s for S. aureus and S. epidermidis were 2- to 4-fold higher (Table 1 and Fig. 1A). In *S.* Typhimurium, MIC_95_s ranged from 14 to 90 μM, depending on the conditions used to compromise the outer membrane (30). These data indicate that JD1 is similarly effective against Gram-positive bacteria and against Gram-negative bacteria that have sustained outer membrane damage.

**TABLE 1:** Concentrations of JD1 that inhibit Gram-positive bacterial growth

Strain	JD1 MIC_50_ (μM)	JD1 cMIC_95_^*a*^	
		μM	μg/ml
B. subtilis ATCC 6633	10.6 ± 6.3	12.5	5.1
S. aureus Newman	35.6 ± 0.5	40.2	16.4
S. aureus FDA209	26.5 ± 1.0	29.7	12.1
S. aureus USA300	29.1 ± 2	41.0	16.7
S. aureus HG001	33.2 ± 4.5	49.0	19.9
S. epidermidis 1457	21.6 ± 2.7	30.8	12.5

a cMIC_95_ is the calculated MIC_95_.

To determine whether JD1 is bactericidal, we monitored optical density at 600 nm (OD_600_) and recovery of CFU in cultures of B. subtilis and S. aureus FDA209 exposed to various concentrations of JD1 (Fig. 1B to E). Within 15 min of treatment at 2× MIC_95_, JD1 reduced the survival of B. subtilis and S. aureus by at least 10-fold and continued to kill cells over the next 2 h. Thus, JD1 is bactericidal for two distinct genera of Gram-positive organisms. While the B. subtilis laboratory strain was more sensitive to JD1 than the pathogenic S. aureus strains, further experiments were carried out with S. aureus due to the propensity of these strains to establish a persister state, form biofilms, and/or replicate within mammalian cells.

### The membrane potential of S. aureus is rapidly disrupted upon JD1 treatment.

**FIG 2:**
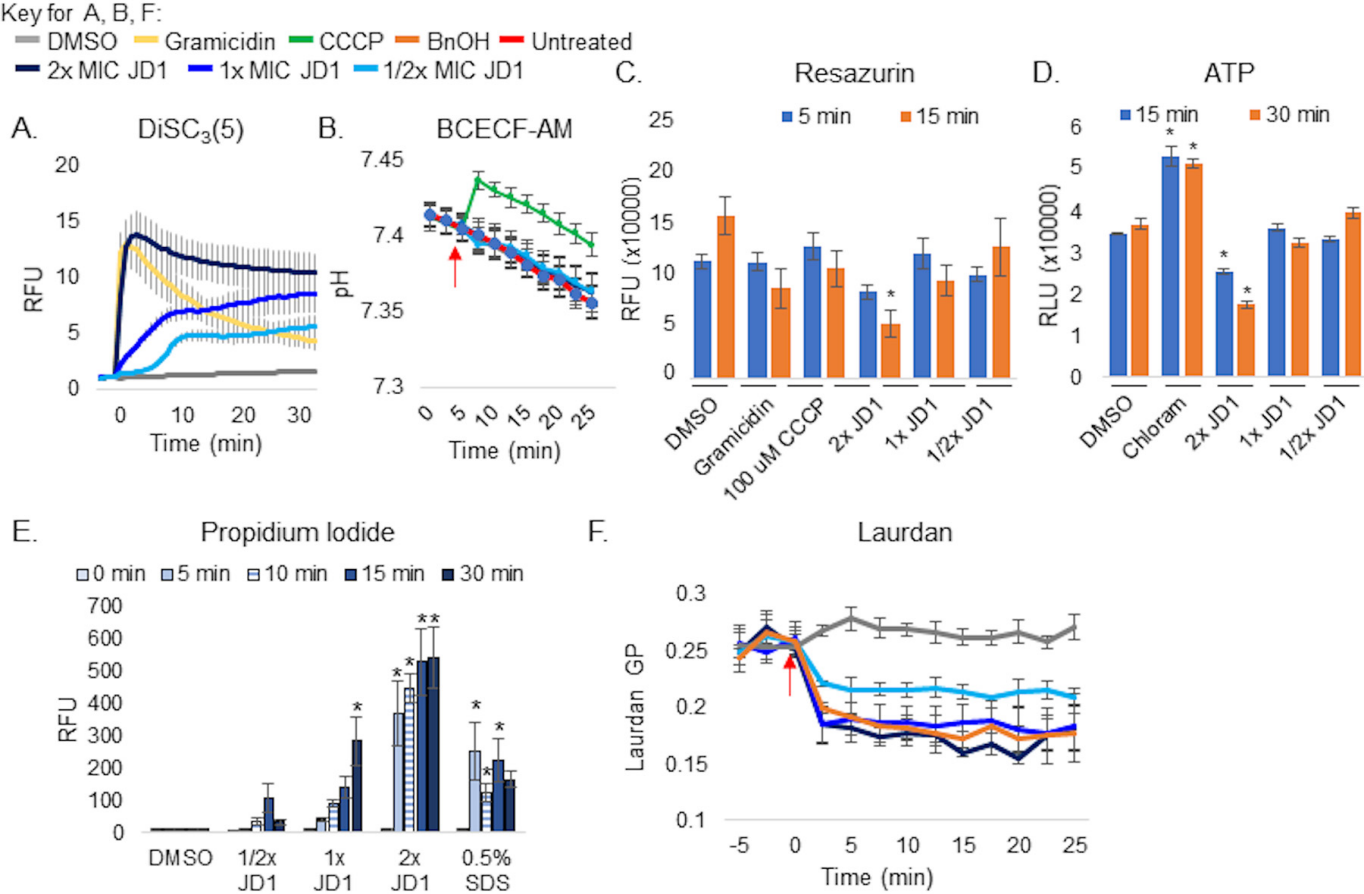
JD1 damages the cytoplasmic membrane of S. aureus FDA209 and reduces laurdan GP without disrupting the pH gradient or respiration. JD1 MIC_95_s are provided in Table 1. Mid-log-phase cells were used for all experiments. (A) Cell membrane potential was monitored with the fluorescent dye DiSC3(5) in LB. Cells were treated at time zero with DMSO, gramicidin (2 μg/ml), or JD1. Means and SEM for three biological replicates performed with technical triplicates are shown. Data are normalized to DMSO at time zero. (B) Intracellular pH was monitored with the fluorescent probe BCECF-AM. Cells were treated with DMSO, the protonophore CCCP (100 μM), or JD1 at the time shown by the red arrow. Means and SEM for three biological replicates performed with technical triplicates are shown. (C) Respiration rates. Cells were incubated with resazurin and treated at time zero with DMSO, gramicidin (8 μg/ml), CCCP (100 μM), or JD1. Means and SEM for three biological replicates performed with technical triplicates are shown. (D) Intracellular ATP levels measured using the Promega BacTiter-Glo kit. Cells were treated for 15 or 30 min with DMSO, chloramphenicol (32 μg/ml), or JD1. Means and SEM for three biological replicates performed with technical triplicates are shown. (E) Cell membrane permeability was monitored by PI fluorescence. Cells were treated at time zero with DMSO, SDS (0.5%), or JD1. Samples were processed at the time points shown. Means and SEM for three biological replicates performed with technical triplicates are shown. (F) Laurdan GP. Cells were treated at the time indicated by the red arrow with DMSO, benzyl alcohol (BnOH) (50 mM), or JD1. Means and SEM for three biological replicates performed with technical triplicates are shown. All asterisks indicate a *P* value of ≤0.05 as determined by one-way analysis of variance (ANOVA) compared to DMSO at that time.

JD1 immediately disrupted the inner membrane and membrane potential of *S*. Typhimurium but much more slowly reduced respiration and ATP accumulation (30). To establish whether the compound acts similarly in S. aureus, cells were loaded with the fluorescent probe 3,3′-dipropylthiadicarbocyanine iodide [DiSC_3_(5)], which accumulates within and is quenched by polarized cell membranes. DiSC_3_(5) becomes highly fluorescent upon release from the membrane (barrier disruption) or depolarization (ion gradients) (37, 38). As anticipated, treatment of S. aureus with gramicidin, a mixture of large (1,882-g/mol), pore-forming peptides that depolarizes membranes (39), increased DiSC_3_(5) fluorescence within minutes (Fig. 2A). When cells were treated with 2× MIC_95_ JD1, an equally rapid and robust increase in fluorescence was observed. At lower doses, the response was slower and more modest. These results show that JD1 disrupts membrane function in S. aureus.

To test whether JD1 affects the pH gradient in S. aureus, we measured the intracellular H^+^ concentration using the fluorescent probe BCECF-AM [2′,7′-bis-(2-carboxyethyl)-5-(and -6)-carboxyfluorescein, acetoxymethyl ester) (40). While treatment with the protonophore carbonyl cyanide *m*-chlorophenyl hydrazone (CCCP) rapidly increased intracellular pH, cells treated with JD1 were indistinguishable from cells treated with DMSO (Fig. 2B), indicating that JD1 does not affect the pH gradient in S. aureus.

We used the indicator resazurin (41) to monitor respiration in S. aureus upon treatment with JD1. A significant decrease in reduction potential was observed only after 15 min in cells treated with 2× MIC_95_ JD1 (Fig. 2C). Similarly, S. aureus exhibited a dose-dependent reduction in ATP levels when treated with 2× MIC_95_ for 15 min (Fig. 2D). These data indicate that the decrease in reduction potential and in ATP levels is likely due to a secondary effect of JD1, suggesting that the primary mechanism of JD1-mediated cell death in S. aureus is membrane barrier disruption.

### Treatment with JD1 perturbs S. aureus membrane barrier function and increases cell membrane fluidity.

In Gram-negative bacteria, JD1 increased the permeability of the cell membrane to the DNA dye propidium iodide (PI). In S. aureus, we similarly found that within 5 min of treatment with 2× MIC_95_ JD1, or with the detergent SDS, there was a significant and dose dependent increase in PI fluorescence compared to DMSO (Fig. 2E). Thus, JD1 rapidly disrupts the barrier function of the cell membrane in S. aureus.

Defects in membrane barrier function may be caused by changes in membrane fluidity (42–44). Laurdan is a fluorescent probe that inserts into the phospholipid bilayer, and its emission spectrum changes with the polarity of its environment, as determined by calculating the generalized polarization (GP). As GP declines, membrane fluidity and/or curvature increases (45–47). Treatment with the membrane fluidizer benzyl alcohol (BnOH) rapidly decreased GP, as expected (48). Treatment with JD1 also decreased GP, in a dose-dependent manner (Fig. 2F). These data are consistent with a rapid JD1 effect that increases the fluidity and/or the curvature of the bacterial cell membrane.

### Abnormal S. aureus membranous structures appear within minutes of JD1 treatment.

**FIG 3:**
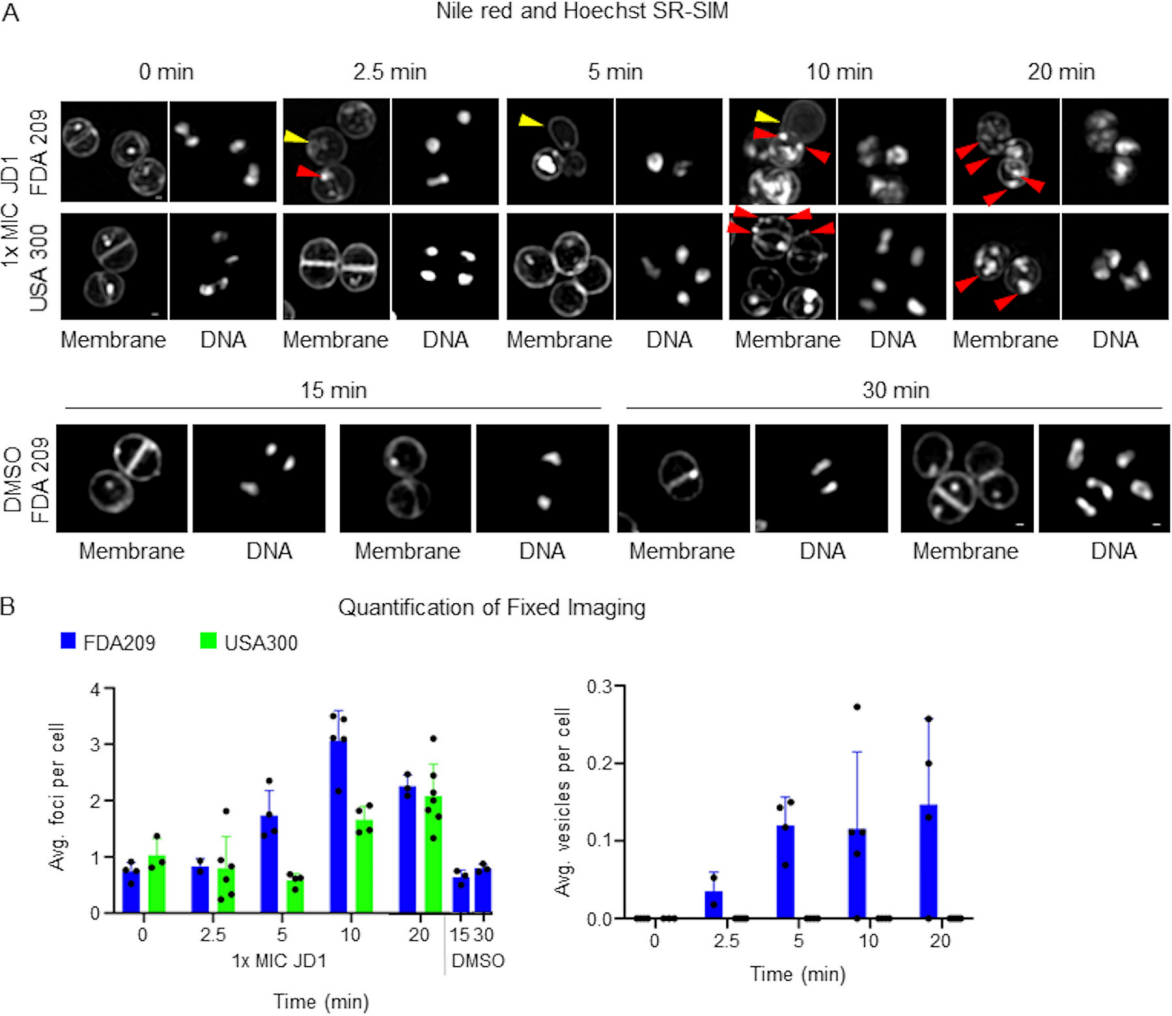
Multiple S. aureus cell membrane aberrations appear in response to JD1 treatment. (A) Nile red and Hoechst staining followed by SR-SIM imaging of S. aureus strains FDA209 and USA300. Cells were grown to mid-log phase, treated with 1× MIC_95_ JD1 (Table 1) or DMSO for the indicated period of time, stained with the lipophilic dye Nile red (30 μM) and the DNA dye Hoechst (30 μM), fixed, and imaged. Yellow arrowheads show examples of Nile red cell membrane blebs or circles inside and outside cells and lacking DNA. Red arrowheads indicate examples of Nile red puncta. Bar, 200 nm. Images are representative of two biological replicates, 2 to 7 fields of view, and 12 to 206 cells. (B) Quantification of aberrations seen in fixed-cell images of S. aureus cells treated with 1× MIC_95_ JD1 (Table 1) or DMSO. Each circle represents the number of puncta per cell in a field of view. Averages and standard deviations (SD) are shown.

Gram-negative bacteria treated with JD1 developed both internal and external membranous blebs, but whether they derived from the inner or outer membrane could not be determined. Since Gram-positive bacteria have only a single membrane, we performed a similar experiment with two S. aureus strains. Cells were treated with 1× MIC_95_ JD1 and stained with the lipophilic dye Nile red and the DNA stain Hoechst 33342. Live imaging suggested rapid membrane perturbation within minutes in the JD1-treated but not the DMSO-treated samples (Fig. S1). JD1 treatment increased the number of Nile red puncta 30-fold over DMSO treatment within 5 min. Fixed-cell imaging enabled higher resolution and revealed that within 2.5 min of treatment with JD1, S. aureus FDA209 cells had accumulated small, Nile red-stained intracellular membranous structures (Fig. 3, yellow arrowheads), and numerous distinct Nile red puncta around the membrane (red arrowheads). After 5 min of treatment, membranous protrusions appeared, apparently lacking DNA, and the number of Nile red puncta increased. Within 10 min, JD1-treated S. aureus acquired a large number of Nile red puncta (Fig. 3; Fig. S1). The accumulation of Nile red patches may represent areas of increased membrane fluidity and/or aberrant structural changes and show that JD1 alters the S. aureus cell membrane within minutes.

10.1128/mBio.01801-21.1 FIG S1 Additional Nile red SR-SIM microscopy. Live-cell imaging of DMSO-treated (A) or JD-1-treated (B) S. aureus FDA209 cells. Montages are representative of two biological replicates. (C) Quantification of puncta per cell. Download FIG S1, TIF file, 0.3 MB. Copyright © 2021 Dombach et al. 2021 Dombach et al. https://creativecommons.org/licenses/by/4.0/ This content is distributed under the terms of the Creative Commons Attribution 4.0 International license.

**FIG 4:**
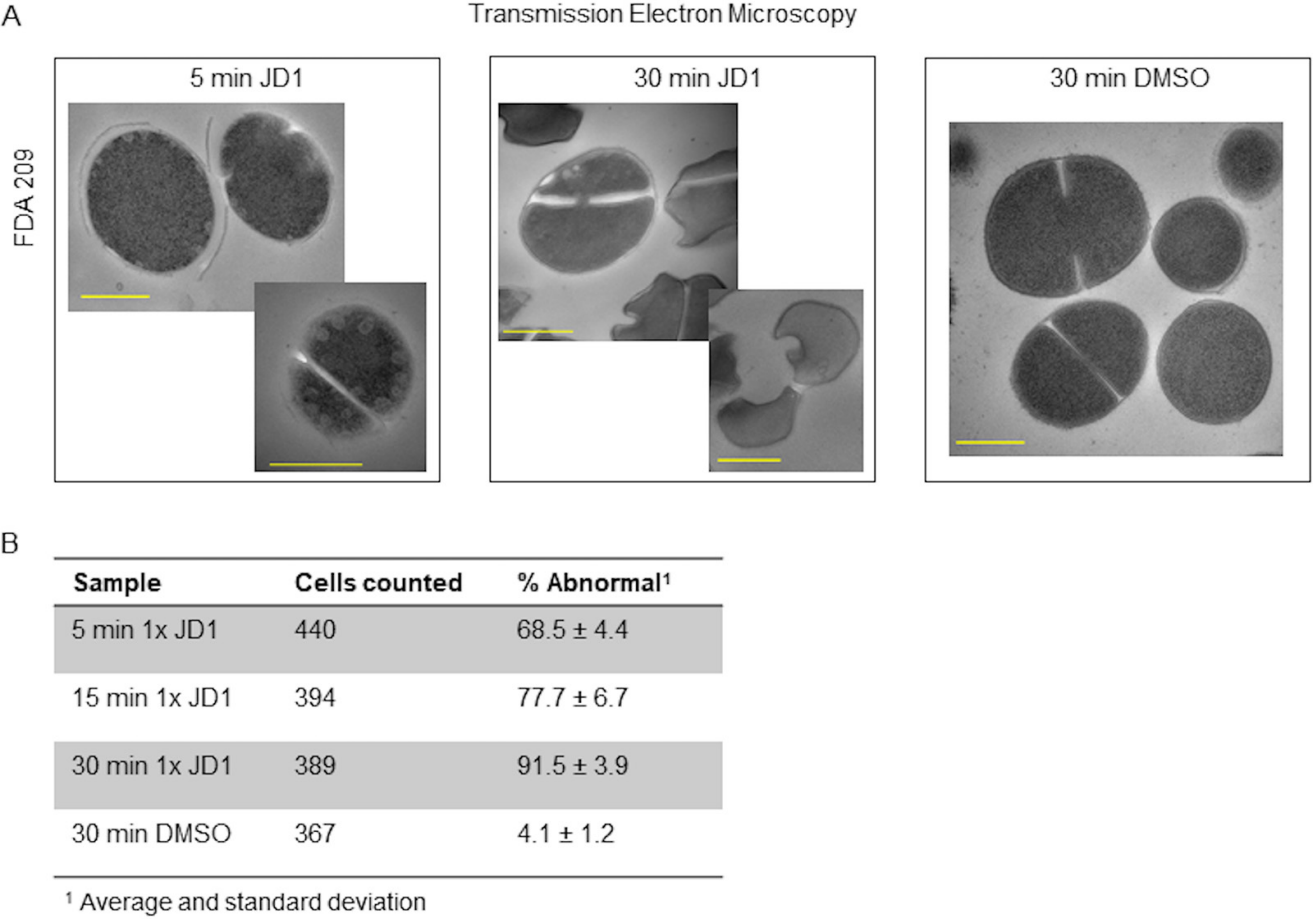
JD1 treatment rapidly leads to abnormal membranous structures in S. aureus. (A) Representative transmission electron micrographs of S. aureus FDA209 treated with 1× MIC_95_ JD1 for 5 and 30 min or with DMSO for 30 min. Bar, 500 nm. Micrographs are representative of biological duplicates. (B) Quantification of TEM images for abnormal S. aureus FDA209 cells when treated with 1× MIC_95_ JD1 or DMSO. A minimum of 103 cells were counted per treatment, per biological replicate.

Since fluorescence microscopy demonstrated localized effects of JD1 on membranes, we investigated whether JD1 altered membranes and/or other cellular structures using transmission electron microscopy (TEM). Cells were treated with 1× MIC_95_ JD1 for 5, 15, or 30 min or DMSO for 30 min and imaged by TEM. Within 5 min of JD1 treatment 68.5% of cells displayed aberrant morphology (Fig. 4; Fig. S2). Most aberrant cells contained at least one intracellular membranous vesicle, with some cells containing eight or more per image. After 15 min of treatment, there was a higher percentage of abnormal cells, many with an undulating membrane and/or membrane separation from the peptidoglycan. While 30 min of treatment with 1× MIC_95_ JD1 was not bactericidal (Fig. 1), almost all of the cells in these samples were abnormal: the septa of many JD1-treated cells were distorted, and the membrane pulled away from the cell wall (Fig. 4A and B; Fig. S2). These data confirm that treatment with JD1 causes rapid formation of internal membranous blebs and further reveal severe cell division and morphological defects.

10.1128/mBio.01801-21.2 FIG S2 Additional TEM micrographs. TEM of cells treated as in Fig. 3B. Lower-power TEM of S. aureus FDA209 treated with 1× MIC_95_ JD1 (Table 1) for the indicated period of time or with DMSO for 30 min. Red boxes indicate cells shown in Fig. 3B. Bar, 500 nm. Micrographs are representative of two biological replicates. Download FIG S2, TIF file, 0.4 MB. Copyright © 2021 Dombach et al. 2021 Dombach et al. https://creativecommons.org/licenses/by/4.0/ This content is distributed under the terms of the Creative Commons Attribution 4.0 International license.

### JD1 decreases the survival of staphylococcal persister cells in broth.

**FIG 5:**
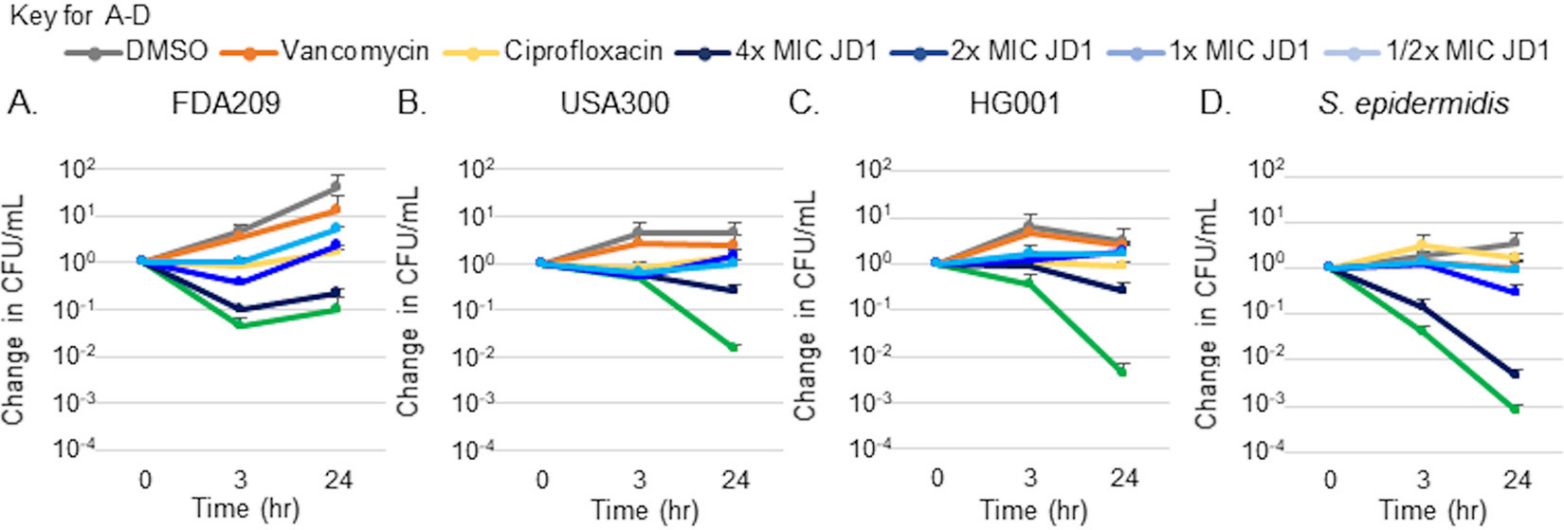
Treatment with JD1 reduced the number of viable staphylococcal persister cells in broth. (A to D) Overnight cultures of the indicated strains were treated at time zero with DMSO, ciprofloxacin (4 μg/ml), vancomycin (10 μg/ml), or the corresponding MIC_95_ of JD1 (Table 1). Cultures were plated for enumeration of CFU at time points indicated. Means and SEM for three biological replicates are shown.

To test whether JD1, like other compounds that target the cell membrane, reduces Gram-positive bacterial persister populations in broth, we established a population of cells with a high percentage of persisters (13, 49). Cultures were grown for 18 h and then treated with DMSO, antibiotics, or a dose range of compound JD1 for 3 or 24 h and then plated for CFU enumeration. Since persister, but not planktonic, cells are resistant to clinical antibiotics such as vancomycin and ciprofloxacin, high doses of these antibiotics were used to ensure that the population of cells being tested were indeed persister cells (11). As expected, in all four strains tested, high doses of vancomycin and ciprofloxacin did not decrease the number of viable cells recovered (Fig. 5A to D). After 24 h of treatment with 2× MIC_95_ JD1, S. aureus FDA209 persister populations decreased 10-fold and S. epidermidis persisters declined 100-fold. Both S. aureus USA300 and HG001 persister populations decreased 100-fold after 24 h of treatment with 4× MIC_95_ JD1 and S. epidermidis persisters decreased 1,000-fold (Fig. 5A to D). These data show that JD1 is particularly potent against persister cells in broth culture.

### Treatment with JD1 does not inhibit Staphylococcus biofilm formation but reduces established biofilms.

**FIG 6:**
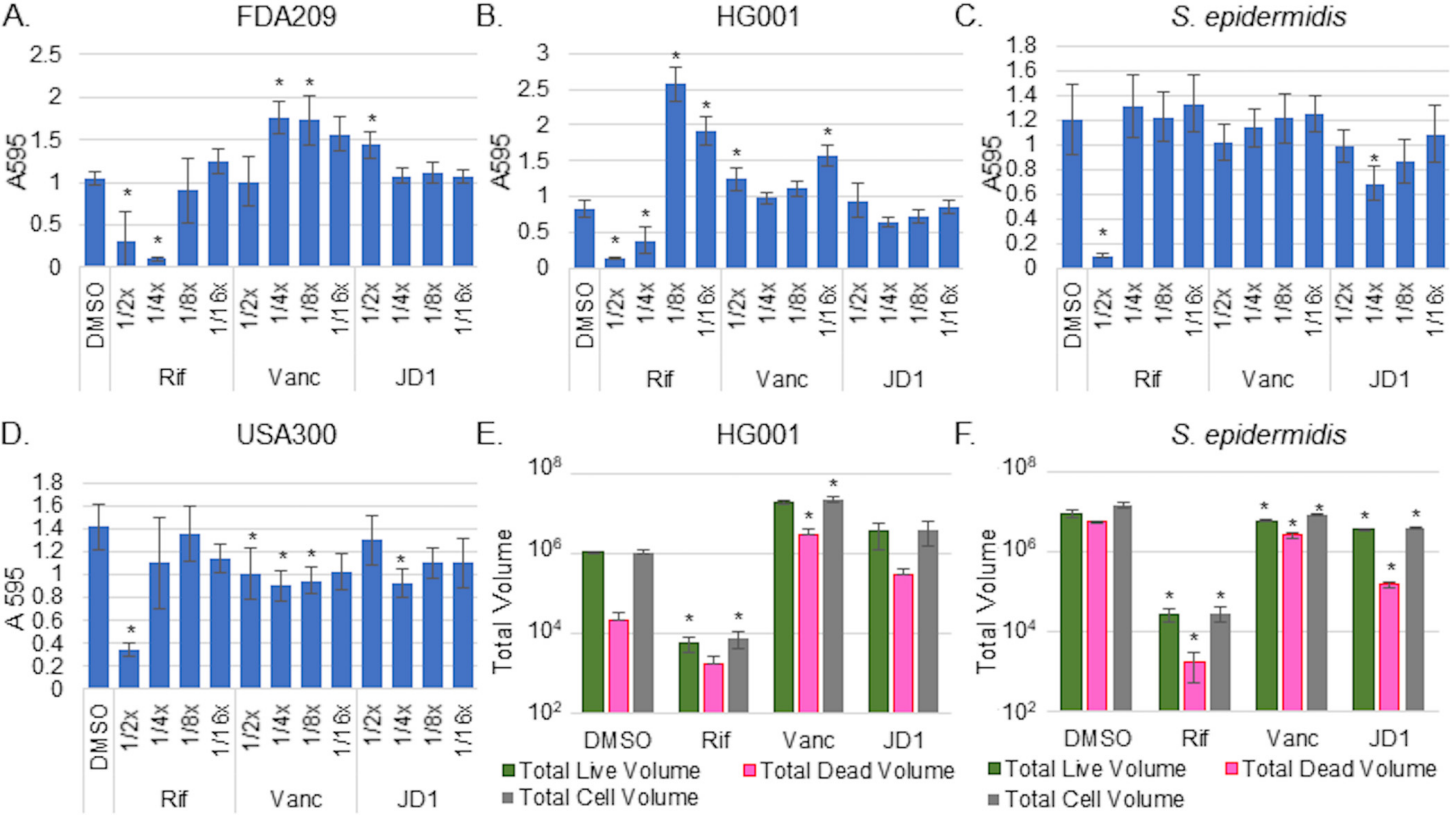
JD1 is minimally effective at inhibiting staphylococcal biofilm formation. Biofilm formation was monitored after 18 h in TSB with subinhibitory concentrations of DMSO, rifampin (1× MIC_95_ = 0.05 μg/ml), vancomycin (1× MIC_95_ = 1 μg/ml), or JD1. (A to D). Crystal violet staining for accumulated biofilm mass measured at *A*_595_. Means and SEM of three biological replicates performed with technical triplicates are shown. (E and F). Biofilms were stained with Syto 9 (live cells) and PI (damaged/dead cells) and imaged with confocal fluorescence microscopy. Volume of live, dead, and total cell volume was calculated (positive volume = positive voxel; 1 voxel = 0.0367 μm^3^). Asterisks indicate *P* values of ≤0.05 as determined by one-way ANOVA compared to DMSO. Panels A to D show means and SEM for three biological replicates performed in triplicate. Panels E and F show means and SDs for one of two biological replicates derived from a minimum of four fields of view.

The bacterial membrane has been posited as an ideal target for preventing and clearing biofilms (24). Since JD1 clearly damages S. aureus cell membranes, we established whether this compound inhibits biofilm formation or maintenance. We evaluated biofilm formation by planktonic S. aureus over 24 h in the presence of JD1 at subinhibitory concentrations, which were necessary to allow S. aureus survival (Fig. 1D). We monitored biofilm mass with crystal violet staining. Both control antibiotics (rifampin and vancomycin) inhibited biofilm formation in some but not all strains tested, and vancomycin promoted biofilm formation in some instances. JD1 treatment slightly inhibited biofilm formation only in S. epidermidis and S. aureus USA300 and promoted biofilm formation in strain FDA209 (Fig. 6A to D). We also monitored the volume of live and dead cells in biofilms using Syto9 and PI, respectively. Biofilms were imaged with a spinning disc confocal microscope, and volumes were quantified. Rifampin treatment significantly reduced the volume of live and dead cells in S. aureus HG001 and S. epidermidis biofilms, but JD1 treatment only slightly inhibited the formation of S. epidermidis biofilms (Fig. 6E and F). JD1 therefore modestly inhibits biofilm formation in some strains tested.

**FIG 7:**
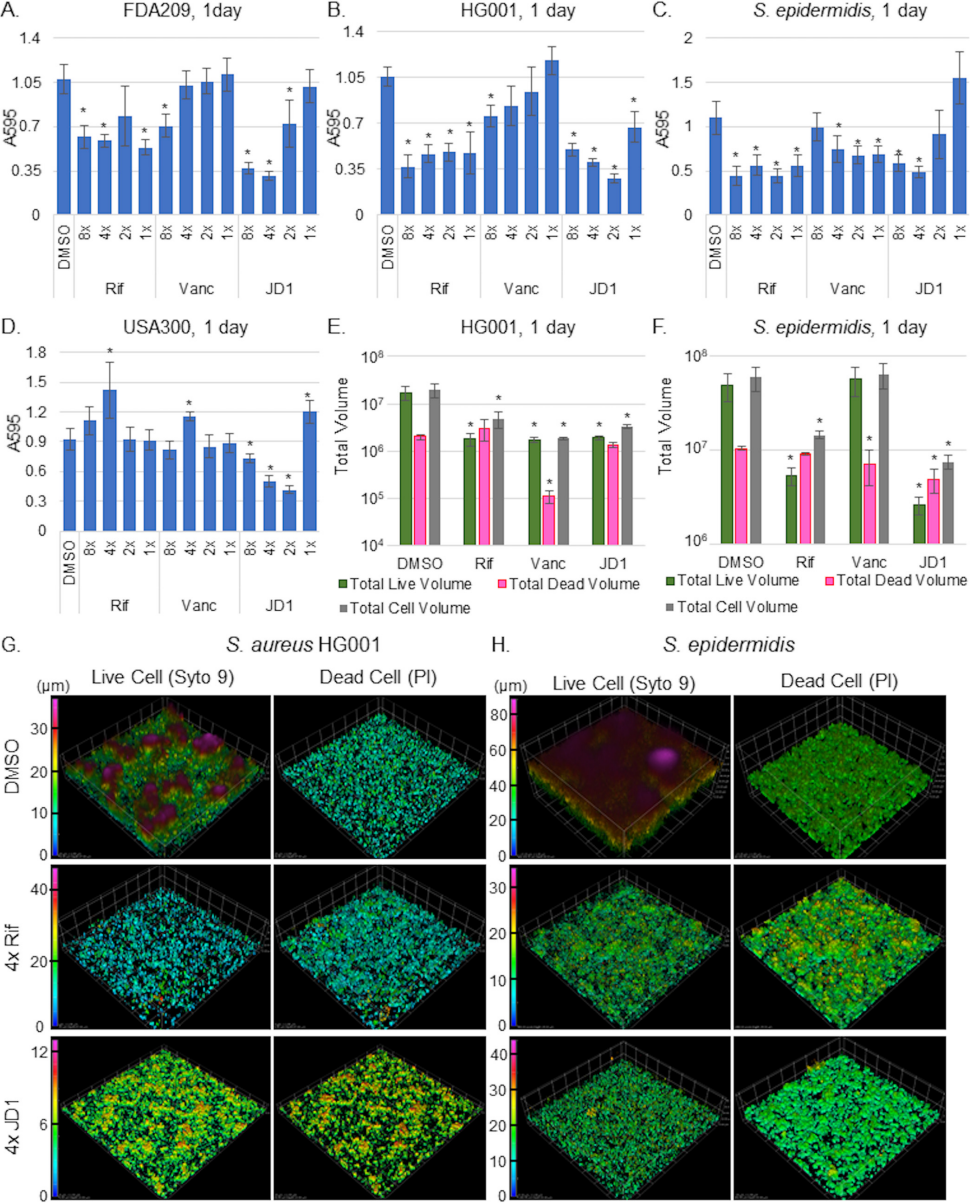
JD1 decreases 1-day-old staphylococcal biofilms. For the indicated strain, biofilms established in TSB for 24 h were treated for 18 h with DMSO, rifampin (1× MIC_95_ = 0.05 μg/ml), vancomycin (1× MIC_95_ = 1 μg/ml), or JD1. The remaining biofilm matrix was quantified with crystal violet (A to D), or volume was quantified after Syto9 and PI staining (E and F) as in Fig. 5G and H) Syto9 and PI-stained Z-stacked images were converted to volumes. Volume data were compiled and reconstructed in Nikon Elements Advanced Research software using a color-coded volume display where blue is the bottom, lowest Z-stack and magenta is the highest point for each sample. The scale is in micrometers. Asterisks indicate *P* values of ≤0.05 as determined by one-way ANOVA compared to DMSO. Panels A to D show means and SEM from three biological replicates performed in triplicate. Panels E and F show means and SDs for one of two replicates derived from a minimum of four fields of view. Panels G and H show representative images from the replicate in E and F.

We next examined whether JD1 disrupts the maintenance of established 1- and 5-day-old biofilms by monitoring mass and volume. We treated biofilms with a range of concentrations of DMSO, vancomycin, rifampin, or JD1 for 18 h. Rifampin reduced day-old biofilms in three of the four Staphylococcus strains tested, and vancomycin less so. However, JD1 reduced the 1-day-old biofilm mass of all Staphylococcus strains tested as well as or better than rifampin (Fig. 7A to D). JD1 was also at least as potent as rifampin at reducing the volume of live cells at 4× MIC_95_ in both staphylococcal species (Fig. 7E and F). Volume reconstructions of the imaged biofilms confirmed that S. aureus HG001 formed shorter (∼38 μm) biofilms than S. epidermidis (∼80 μm). Nevertheless, treatment of either strain with JD1 reduced biofilm height by decreasing the volume of live cells, as opposed to decreasing the volume of dead cells (Fig. 7G and H). In addition, the reconstructions of the DMSO controls for S. aureus HG001 revealed peaks of biofilm that were diminished by JD1 or rifampin treatment (Fig. 7G). There was no significant JD1 effect on 5-day-old biofilms, with the exception that 8× MIC_95_ JD1 reduced the mass of S. aureus FDA209 (Fig. S3). These data illustrate that JD1 was effective at reducing the mass and cell volume of 1-day-old biofilms in all strains tested as well as or better than antibiotic controls.

10.1128/mBio.01801-21.3 FIG S3 JD1 is minimally effective at reducing 5-day-old staphylococcal biofilms. (A to D) For the indicated strain, biofilms established in TSB for 5 days were treated for 18 h with DMSO, rifampin (1× MIC_95_ = 0.05 μg/ml), vancomycin (1× MIC_95_ = 1 μg/ml), or JD1 (Table 1). Remaining biofilm extracellular matrix was stained with crystal violet, and the *A*_595_ was measured. Means and SEM for three biological replicates performed with technical triplicates are shown. Asterisks indicate *P* values of ≤0.05 as determined by one-way ANOVA compared to DMSO. Download FIG S3, TIF file, 0.05 MB. Copyright © 2021 Dombach et al. 2021 Dombach et al. https://creativecommons.org/licenses/by/4.0/ This content is distributed under the terms of the Creative Commons Attribution 4.0 International license.

### JD1 reduces replication and survival of intracellular S. aureus.

**FIG 8:**
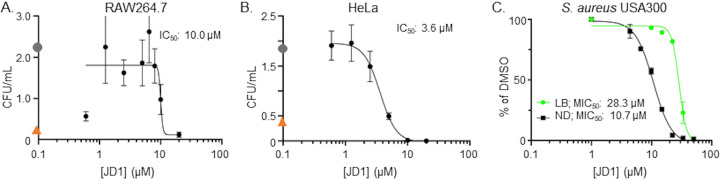
Treatment with JD1 reduces S. aureus survival within mammalian cells. (A) RAW 264.7 macrophage-like cells or (B) HeLa cells were infected with S. aureus USA300 at MOIs of 0.5 and 2.5, respectively. Cells were treated 2 h after infection with DMSO (gray circle), vancomycin (25 μg/ml; orange triangle), or dilutions of JD1 from 20 μM. After 8 h of infection, cells were lysed and plated for enumeration of CFU. Means and SEM of biological duplicates performed with technical triplicates, each with six dilutions of JD1, are shown. The IC_50_s are indicated. (C) Dose-response curves monitoring bacterial growth of S. aureus USA300 from an OD_600_ of 0.01 in nutrient-depleted (ND) media and LB normalized to growth in 2% DMSO. Means and SEM for at least three biological replicates performed with technical triplicates are shown. All data are from at least biological triplicates for each concentration of JD1. (A and B) Numbers are 10^5^ CFU/ml for A and B.

Since intracellular S. aureus may be a major cause of reoccurring infections and contribute to the development of antibiotic resistance, we next tested whether JD1 was effective at reducing viable intracellular S. aureus with the USA300 strain in both RAW264.7 and HeLa cells (50, 51). As a control, infected cells were treated with vancomycin at a concentration (25 μg/ml;16.83 μM) within the range found in the blood of patients treated with this last line of defense antibiotic (52). Vancomycin decreased the number of recoverable bacteria less than 10-fold. JD1 treatment decreased the number of recoverable bacteria in both cell types to the limit of detection, with a 50% inhibitory concentration (IC_50_) of 10.0 ± 1.1 μM in RAW264.7 cells and of 3.6 ± 1.3 μM in HeLa cells (Fig. 8). Thus, JD1 is more potent against S. aureus within host cells than in broth culture. Since bacteria within host cells are exposed to lysozyme and other innate immune insults, we tested whether lysosome potentiates JD1 but found no evidence of interaction (Fig. S4). We also examined whether growth in nutrient-limited medium increased the potency of JD1, as host cells often limit pathogen access to nutrients. The MIC_50_ of JD1 in S. aureus USA300 under nutrient-limiting conditions was 10.7 ± 0.6 μM, compared to 29.1 ± 2.0 μM in LB (Fig. 8C), suggesting that nutrient limitation potentiates JD1. Moreover, at concentrations that are not toxic to host cells (30), JD1 reduced the survival of intracellular S. aureus USA300 better than vancomycin.

10.1128/mBio.01801-21.4 FIG S4 JD1 does not appear to synergize with lysozyme in broth culture. S. aureus FDA209 in LB was grown overnight in the presence of JD1 and lysozyme. The darkest orange represents an OD_600_ of 1.05, and white represents 0.0. Download FIG S4, TIF file, 0.02 MB. Copyright © 2021 Dombach et al. 2021 Dombach et al. https://creativecommons.org/licenses/by/4.0/ This content is distributed under the terms of the Creative Commons Attribution 4.0 International license.

## DISCUSSION

### Mechanism of action of JD1 in Staphylococcus species.

The small molecule JD1 enables the killing of *S.* Typhimurium in macrophages and damages Gram-negative inner membranes when the outer membrane is permeabilized or in strains with loss-of-function mutations in efflux pumps. Here, we examined the effect of JD1 on Gram-positive bacteria, which lack an outer membrane. JD1 was bactericidal in two genera of Gram-positive bacteria and appeared to damage the cell membrane of S. aureus. Upon exposure to JD1, the membrane rapidly depolarized, laurdan GP decreased, and intracellular membranous blebs were observed. Within 5 min, barrier function was disrupted and numerous intracellular vesicles had accumulated within a majority of cells. The rapid changes observed suggest that JD1 affects the arrangement of lipids; decreased laurdan GP is consistent with either or both increased membrane fluidity or the curvature of internal membrane vesicles (45, 47, 53). With longer periods of exposure to JD1, ATP levels and reduction potential declined. JD1 also killed persister cells, reduced the matrix and cell volume of 1-day-old biofilms, and increased the killing of intracellular S. aureus. Thus, the cell membrane-damaging activity of JD1 enables this compound to prevent the survival of S. aureus grown under conditions germane to infection.

### Comparison of JD1 and vancomycin activity against Staphylococcus species.

Vancomycin (1449 g/mol) inhibits cell wall biosynthesis and is currently used as a last line of defense drug against methicillin-resistant S. aureus (MRSA) (54). Vancomycin is extremely effective at killing S. aureus in broth culture, with an MIC_95_ in LB of ∼1 to 2 μg/ml, approximately 15-fold less than that of JD1. However, when difficult-to-treat growth stages are considered, JD1 is more effective than vancomycin (Table 2). Vancomycin was not able to kill staphylococcal persister cells in broth culture, whereas JD1 reduced persisters by as much as 1,000-fold. JD1 was also more effective than vancomycin at reducing 1-day-old staphylococcal biofilms and viable intracellular S. aureus. These data underscore the importance of testing compound potency not only in planktonic bacteria but also under infection-relevant growth conditions. In addition, compounds that damage cell membranes may be more useful for treating critical *in vivo* staphylococcal growth stages than clinical antibiotics.

**TABLE 2:** Comparison of vancomycin and membrane-active small molecules against different types of staphylococcal growth

Growth state	Relative potency^*a*^				
	JD1	Vancomycin	Violacein	Rhodomyrtone	Reutericyclin
Broth culture	+	+++	+++	+++	++
Persisters	++	−	NA	++	NA
Biofilms	++	+	+++	++	+++
Intracellular infection	+++	+	NA	+	+

a Relative potency of JD1 versus the indicated compounds. The minus sign indicates no detected potency. NA, not applicable (the corresponding experiments were not done or the results were not clear).

### Utility of JD1 for establishing membrane vulnerabilities of Gram-positive bacteria in different growth states.

The observation that planktonic cells are vulnerable to membrane-damaging agents such as JD1 is consistent with previous results obtained with other bactericidal small molecules (Table 2). For instance, violacein (343 g/mol), like JD1, disrupts membrane barrier function and causes rapid ATP leakage in planktonic cells (55, 56). In contrast to JD1, neither rhodomyrtone (443 g/mol) nor reutericyclin (349 g/mol) disrupts barrier function. Rhodomyrtone, like JD1, causes rapid membrane depolarization (57). Reutericyclin dissipates the pH gradient (58, 59). Thus, planktonic Gram-positive cells are susceptible to multiple kinds of membrane damage.

The properties of JD1 further reveal the utility of this compound for identifying the vulnerabilities of persister cells, biofilms, and intracellular bacteria (Table 2). Rhodomyrtone, like JD1, probably kills persisters, as it reduces viable cells in a nongrowing culture by 4 orders of magnitude (57). Therefore, the energetics of persister cell membranes are likely susceptible to small molecules. Given that biofilms are inherently difficult to treat, it is encouraging that JD1, rhodomyrtone, violacein, and reutericyclin all have potency against biofilms (23, 60–62). These data indicate that agents with distinct membrane-damaging activities will be useful as tools to clarify the weak points of biofilms. However, whether any of these compounds, like JD1, kill intracellular bacteria remains unknown.

### Infection potentiates small-molecule access to Gram-positive bacterial cell membranes.

During infection, soluble host defense molecules damage the cell wall of Gram-positive bacteria, likely increasing their sensitivity to compounds. For example, the S. aureus USA300 strain is exposed to harsh conditions during replication within the macrophage phagolysosome (50), including various antimicrobial peptides (AMPs), reactive oxygen species (ROS), proteases, lysozyme, low pH, and limited nutrients (63–65). S. aureus responds by producing enzymes that detoxify ROS (66, 67), generating ammonia to counter acidification (68), or modifying the membrane and peptidoglycan to resist AMPs and lysozyme (69–71). In HeLa cells, S. aureus USA300 escapes the phagosome and replicates in the host cell cytoplasm (72, 73). In this study, we found that S. aureus is 2 to 3 times more sensitive to JD1 in HeLa cells with respect to the IC_50_ and the reduction of viable bacteria than in RAW264.7 cells, possibly reflecting bacterial physiological differences in these two host cell types. Furthermore, S. aureus was more sensitive to JD1 in host cells than in LB and had heightened sensitivity to JD1 in nutrient-depleted medium versus LB. These data suggest that nutrient limitation within host cells increases S. aureus vulnerability to membrane damage. The multiple environmental stresses to which bacteria are exposed within an animal act in concert to cripple bacteria, and dynamic infection microenvironments are not likely duplicated in any broth culture.

### Host membrane toxicity of small membrane-targeting probes.

A key question for all membrane-active compounds is whether their activity is specific to bacterial versus mammalian cell membranes. This issue is generally addressed with hit compounds from screens, but the interpretation of results is complicated. First, if membrane-targeting compounds interact with host cell membranes, the much larger volume of host membrane likely dilutes out the compound, reducing the concentration that reaches the bacteria. While mammalian cell cholesterol and neutral lipids appear to protect host cells from JD1 (30), we do not know how much JD1 reaches bacteria in the phagosome. However, since *S.* Typhimurium in macrophages is killed by JD1 at concentrations that are 30-fold lower than that which disrupt host cell membranes, *S.* Typhimurium in phagosomes likely becomes vulnerable to small molecules due to cell envelope damage caused by host innate immunity (30, 74, 75). Both JD1 and rhodomyrtone are probably too toxic for development as therapeutics; they kill cultured cells at concentrations as low as 10 μM and 1 to 3 μM, respectively (30, 76). However, analogs of reutericyclin are 12 times less toxic to host cells and still damage bacterial cell membranes (58), demonstrating that medicinal chemistry efforts may separate compound toxicity from activity.

### Conclusions.

Collectively, JD1 and the other small molecules described suggest the feasibility of selective targeting of the bacterial cell membrane over host membranes. They also reveal that membrane-active compounds are effective against MDR bacteria and that some are potent against persister cells, biofilms, and intracellular bacteria. The variability in compound potency under different growth conditions and within mammalian cells highlights the importance of identifying and optimizing compounds under conditions relevant to infection. Moreover, the cell membranes of both Gram-negative and Gram-positive bacteria may be useful antibiotic targets for difficult-to-treat infections.

## MATERIALS AND METHODS

### Bacterial strains.

B. subtilis (ATCC 6633), S. aureus Newman (AH1178), S. aureus FDA209 (ATCC 6538), S. aureus HG001 (AH2183), S. aureus USA300, and S. epidermidis 1457 (AH2490) were used in this study.

### Media and reagents.

Unless otherwise stated, bacteria were grown in lysogeny broth (LB) at 37°C with aeration. JD1 is commercially available (BTB12794; MolPort). To obtain mid-log-phase cells, bacteria were grown overnight in LB, diluted the next morning 1:100 in fresh LB, and then grown to mid-log phase (OD_600_, 0.4 to 0.6). To test bacterial growth in nutrient-depleted conditions, bacteria were grown in 0.25× SSM9PR (0.25× M9 salts, 0.5 mM MgSO_4_, 0.25 mM CaCl_2_, 0.25% glucose, 0.25% Casamino Acids, 0.25 mM thiamine-HCl, 12.5 μM nicotinamide). The fractional inhibitory concentration indexes (FICIs) for JD1 and novobiocin in *S.* Typhimurium (SL1344) (77) and E. coli (K-12) (78) were established in M9 (42 mM Na_2_HPO_4_, 22 mM KH_2_PO_4_, 18.7 mM NH_4_Cl, 8.5 mM NaCl, 0.1% Casamino Acids, 1 μM MgSO_4_, 2% glucose) (30) and calculated as follows (79): (MIC_DrugA in combination_/MIC_DrugA alone_) + (MIC_DrugB in combination_/MIC_DrugB alone_).

### MIC determination.

Overnight cultures were diluted in LB to an optical density at 600 nm (OD_600_) of 0.01 and distributed into polystyrene 96-well flat-bottom plates (Greiner; 655185). Compound was added to the desired final concentration, and the final DMSO concentration never exceeded 2%. Plates were grown at 37°C with shaking for 18 h, and OD_600_ was monitored (BioTek Synergy H1 or BioTek Eon). MICs were defined as the concentration at which 95% of growth was inhibited (determined by OD_600_) using the curve determined by a variable slope nonlinear regression in GraphPad Prism with a 95% confidence interval.

### Growth curves and kill curves.

Mid-log-phase cultures were sampled at time zero, and then compound or vehicle control (DMSO) was added. Cultures were incubated at 37°C with agitation. At the time intervals indicated, aliquots were monitored for OD_600_ and plated for CFU enumeration. Data for OD_600_ and CFU/ml were normalized to those at time zero.

### Membrane potential assays.

Membrane potential was measured using the potentiometric fluorescent probe DiSC_3_(5) (Invitrogen). Mid-log-phase cells were diluted to an OD_600_ of 0.4. DiSC_3_(5) was added to a final concentration of 2 μM and the culture was incubated at 37°C in a rotator for 15 min. To remove unincorporated DiSC_3_(5) remaining in the medium, cells were captured with vacuum filtration on a 0.45-μm Metricel membrane filter (Pall), resuspended in fresh LB, and distributed (200 μl) into black polystyrene 96-well plates (Greiner, 655076). Plates were monitored (excitation wavelength [ex], 650 nm; emission wavelength [em], 680 nm) on a BioTek Synergy H1 plate reader. After baseline fluorescence was recorded, compound was added to the desired final concentration, and measurements were recorded for an additional 30 min. Control wells without bacteria and containing medium with 2 μM DiSC_3_(5) and DMSO had an average RFU of 3,197 ± 179. After the addition of 2×, 1× (30 μM for S. aureus FDA209), or 0.5× JD1, DiSC_3_(5) fluorescence was 191% ± 9%, 169% ± 6.9%, and 144% ± 3.3% of that in DMSO-treated cells, respectively. These values represent the maximum increase in fluorescence due to compound addition in the presence of DiSC3(5). Note that with 2× MIC JD1 in the presence of cells (Fig. 2A), DiSC_3_(5) signal increased by approximately 15,000%, dwarfing the percent increase observed without cells present.

### Monitoring intracellular pH with BCECF.

BCECF-AM (Molecular Probes) was added to mid-log-phase cells in modified LB (LB with 0.1% glucose, 50 mM HEPES, 300 mM KCl [pH 7.0]) to a final concentration of 10 μM and incubated at 37°C in a rotator for 1 h. Cells were diluted 1:10 and pipetted into a black polystyrene 96-well plate (Greiner; 655076). After 5 min of equilibration, compounds were added and fluorescence (ex, 490 nm/em, 535 nm; ex, 440 nm/em, 535 nm) was monitored every 2.5 min for 20 min using a BioTek Synergy H1 plate reader. BCECF fluorescence was calibrated at 7 pHs between 5.5 and 8 (0.5 pH increments): pH = pK_a_ − log(*I*_490_/*I*_440_) (where *I*_490_ and *I*_440_ are the fluorescence intensities at 490 and 440 nm, respectively); the pK_a_ of BCECF is 6.97 (80). During calibration with untreated cells, fluorescent signal did not change in cells incubated in media at pH 5.5. In contrast, signal declined over time in media at pH 8.0, as *I*_440_ decreased and *I*_490_ increased. Thus, the decline in signal observed across all treatments likely reflects cell acclimation to pH 7.

### Resazurin assays.

Mid-log-phase cells (200 μl per well) were transferred to a black polystyrene 96-well plate (Greiner; 655076) containing compound. Compound was added, and the plate was incubated with shaking. Resazurin (alamarBlue; Invitrogen) was added to a final concentration of 100 μg/ml 5 min prior to the indicated time point. The plate was incubated with shaking in the dark at room temperature for 5 min. Fluorescence readings were taken (ex, 570 nm/em, 650 nm) using a BioTek Synergy H1 plate reader.

### ATP measurements.

Intracellular ATP levels were measured using a BacTiter-Glo microbial cell viability assay (Promega) according to the manufacturer’s instructions. Mid-log-phase cells (100 μl) were added to DMSO, chloramphenicol (32 μg/ml) (81), or 2 μl of compound in a black polystyrene 96-well plate (Greiner; 655076) and incubated for 10 or 25 min at 37°C with agitation. Reagent (100 μl) was added, and samples were incubated in the dark with agitation for 5 min. Luminescence was read on a BioTek Synergy H1 plate reader.

### Propidium iodide membrane barrier assays.

Compound, DMSO, or SDS (0.5%) was added to mid-log-phase cells to the desired concentration, and cultures were sampled at 0, 5, 10, 15, and 30 min. Five minutes before harvesting, PI [10 μg/ml] (Life Technologies) was added. Cells were pelleted, washed twice, resuspended in phosphate-buffered saline (PBS), and monitored (ex, 535/em, 617 nm) using a BioTek Synergy H1 plate reader.

### Membrane fluidity assays with laurdan.

Cells were grown to mid-log phase in LBg (LB with 0.2% glucose). Laurdan (Invitrogen) was added to a final concentration of 10 μM and incubated at 37°C with rotation for 30 min. Cells were harvested by centrifugation, washed three times, and resuspended in prewarmed PBSg (PBS with 0.2% glucose). Cells (200 μl) were transferred to a black polystyrene 96-well plate (Greiner; 655076) and monitored (ex, 360/em, 450 and 500 nm) on a BioTek Synergy H1 plate reader. Baseline fluorescence was recorded for 5 min prior to addition of compound. Fluorescence was recorded for 25 additional minutes. Laurdan generalized polarization (GP) was calculated: GP = (*I*_460_ − *I*_500_)/(*I*_460_ + *I*_500_).

### Bacterial SR-SIM fluorescence microscopy.

Nile red (Sigma-Aldrich) and Hoechst 33342 (Sigma-Aldrich) were added to mid-log-phase cells to a final concentration of 30 μM and incubated at 37°C for 10 min. For live-cell imaging, cells were harvested by centrifugation at 10,000 × *g* for 1 min and resuspended in 100 μl FluoroBrite Dulbecco’s modified Eagle medium (DMEM). Three microliters of cells was deposited onto an agar pad (20% LB, 2% agarose) containing DMSO or 1× MIC JD1. Cells were covered with a number 1.5H coverslip and imaged at the indicated times as described below. For fixed-cell imaging, cells were treated with DMSO or 1× MIC JD1 for the time indicated. Cells were harvested by centrifugation at 10,000 × *g* for 1 min. The supernatant was carefully aspirated off, and the cells were fixed for 10 min in 1 ml of 4% EM-grade paraformaldehyde (PFA; Electron Microscopy Sciences) diluted in PBS. Cells were harvested by centrifugation at 10,000 × *g* for 1 min and resuspended in 100 μl of PBS, and 20 μl was placed on a number 1.5H glass coverslip. Bio-Rad Frame-Seal incubation chambers (15 by 15 mm, 65 μl) were adhered to glass slides and filled with 30 μl of PBS. Coverslips were inverted and adhered to the Frame-Seal chambers, creating a sealed container with 50 μl of cell suspension. For both live-cell and fixed-cell imaging, slides were imaged in 3D-SIM mode using a Nikon structured illumination superresolution microscope with a 100×/1.49 numerical aperture (NA) oil SR Apo TIRF WD 0.12 (mm) objective and/or an iXon X3 EM-CCD 512-by-512 16-bit camera. Standard filter sets were used to capture Hoechst and Nile red emissions, with excitation at 405 and 561 nm, respectively. Nikon Perfect Focus and manual focusing were used to find the best focal plane during acquisition. Images were reconstructed using Nikon Elements SR-SIM analysis software with the default reconstruction parameters.

### Electron microscopy.

Mid-log cultures were treated with JD1 or DMSO for the stated amount of time and centrifuged at 10,000 × *g* for 2 min, and most of the supernatant was removed. Pelleted samples were resuspended in cryoprotectant (150 mM d-mannitol in growth medium) and then centrifuged to a loose pellet, and supernatant was discarded. A few microliters of each pelleted sample was high-pressure frozen using a Wohlwend Compact 02 high-pressure freezer (Technotrade International, Manchester, NH) as described previously (82). Frozen specimens were then freeze-substituted in anhydrous acetone containing 2% osmium tetroxide and 0.2% uranyl acetate and embedded in Epon/Araldite resin. Serial thin sections (60 to 80 nm) were cut using a Leica UCT ultramicrotome. The serial sections were collected on Formvar-coated copper slot grids, poststained with 2% aqueous uranyl acetate followed by Reynold’s lead citrate, and imaged using a Tecnai T12 Spirit TEM, operating at 100 kV. Thin sections (80 nm) were cut using a Leica Ultracut UCT microtome and collected onto Formvar-coated copper slot grids. The sections were poststained with 2% uranyl acetate and Reynold’s lead citrate. Samples were imaged in a Tecnai T12 SpiritBT TEM using an AMT charge-coupled device (CCD) camera.

### Persister assays.

Cultures were grown overnight in Trypticase soy broth (TSB) at 37°C with aeration for 18 h and then divided into samples. Each sample was monitored for enumeration (time zero), and then antibiotic or compound was added to the desired final concentration. Samples were incubated at 37°C with aeration and monitored at 3 and 24 h for enumeration.

### Biofilm inhibition assays.

Cultures were grown to mid-log phase in TSB and diluted to 4 × 10^6^ CFU/ml in TSB, and 200 μl of this dilution was added to each well of a flat-bottomed polystyrene 96-well (Greiner; 655185) plate; 4 μl of DMSO, antibiotic, or compound was added to achieve the desired final concentration. Edge wells on the plate were filled with PBS to minimize evaporation of experimental wells. After 24 h of incubation at 37°C with no agitation, the media and planktonic cells were carefully removed, and wells were washed twice with PBS. A multichannel pipette was used for removal of media and subsequent PBS washes.

### Biofilm growth and treatment.

Cultures were grown to mid-log phase in TSB and diluted to 4 × 10^6^ CFU/ml in TSB, and 200 μl was added to each well of a flat-bottomed polystyrene 96-well (Greiner; 655185) plate (edge wells on the plate were filled with PBS to minimize evaporation of experimental wells). After 24 h of incubation at 37°C without agitation, wells were carefully washed twice with PBS followed by the addition of 200 μl of TSB containing 4 μl DMSO, antibiotic, or compound to achieve the desired final concentration. For 5-day biofilm assays, wells were washed and received fresh media daily, and DMSO, antibiotic, or compound was added on day 5. Plates were incubated at 37°C for 18 h without agitation. Prior to staining, the biofilm was washed twice with PBS to remove nonadherent cells.

### Measuring biofilm mass with crystal violet.

Plates containing washed biofilms were allowed to air dry; then, 200 μl of 0.01% crystal violet was added to each well and incubated for 20 min. The biofilms were washed once with deionized (DI) water and air dried. The stained biofilm was resuspended in 200 μl 70% ethanol, and the *A*_595_ was measured (BioTek Synergy H1 or BioTek Eon).

### Biofilm volume microscopy and analysis.

Biofilms were grown as described above, with the following differences. Biofilms were grown in Cellvis 35-mm number 1.5H glass-bottom, 20-mm-well dishes. PBS washes to remove nonadherent cells (cells not attached to the biofilm) were performed with serological pipettes to minimize disruption. Biofilms were stained for 15 min prior to imaging with PI (10 μg/ml) and Syto9 (3 μM) resuspended in PBS. The dye solution was gently removed with a pipette, and samples were washed twice with PBS and imaged live (fixation with 4% PFA resulted in a PI-staining artifact). Images were acquired using the Yokogawa CellVoyager CV1000 confocal scanner system with a 40×/0.6 NA working distance 2.7 to 4.0 (mm) objective, a Microlens-enhanced Nipkow disk scanner with a pinhole size of 50 μm, and a Hamamatsu Photonics ImagEM X2 EM-CCD C9100-14 camera with high-resolution 16-bit format. Prior to image acquisition, the Z range of each biofilm was determined by manually establishing the top and bottom, and the z-step size was set to 1 μm to simplify volume analysis (conversion between volume and voxels). Images were acquired in two color channels: ex/em for Syto9 and PI at 488 nm_525/50 and 561 nm_617/73, respectively. A minimum of eight randomly selected fields of view were acquired, and at least four images were used for analysis.

Images were imported into MATLAB R2020b as multidimensional tiff stacks and processed as volume data using a custom script designed to store all results in a data structure for user review. Otsu’s method was used instead of manual thresholding due to different levels of background signals across samples (83). Three-dimensional (3D) binary masks were created to extract Syto9 and PI foreground signals from background (83). The total volume for each of the two channels was quantified as the summation of voxels identified above the threshold per channel. The total number of voxels was converted to total volume (1 voxel = 0.0367 μm^3^) using the metadata. Selected volumes were reconstructed in Nikon Elements Advanced Research software using thresholds determined by Otsu’s method (from the MATLAB script) to provide a color-coded quantitative volume display based on depth in micrometers.

### Intracellular infection assays.

RAW 264.7 (TIB-71) macrophages (5 × 10^4^ macrophages in 100 μl of complete DMEM) were seeded in 96-well tissue culture plates (Greiner; 655180) and were incubated at 37°C with 5% CO_2_. For experiments performed with HeLa cells (ATCC CCL-2), 1 × 10^4^ cells were seeded. S. aureus USA300 was grown overnight in TSB, subcultured to an OD_600_ of 0.4 in TSB, regrown to an OD_600_ of 0.6, and diluted to a final concentration of 5 × 10^5^ CFU/ml in complete DMEM. Twenty-four hours after seeding, 50 μl of bacterial culture was added to each cell culture well, an approximate multiplicity of infection (MOI) of 0.5 bacterium to one cell for RAW 264.7 cells and 2.5 bacteria to one HeLa cell. Plates were centrifuged at 500 × *g* for 2 min to synchronize the infection. Thirty or 45 min (for RAW 264.7 or HeLa cells, respectively) after bacterial addition, wells were washed once with PBS, DMEM containing 100 μg/ml gentamicin (Sigma-Aldrich) was added, and cells were incubated for 90 min. Wells were washed twice with PBS. Complete DMEM was added to the wells, followed by 1 μl of JD1, vancomycin, or vehicle control to the stated final concentration. The highest concentration of JD1 tested was 20 μM, because higher concentrations were toxic to cells (30). IC_50_s and 95% confidence intervals were determined using a variable slope nonlinear regression in GraphPad Prism.

### Data availability.

MATLAB scripts are freely available via the MATLAB file exchange and GitHub (J.L.J.Q.).
