# Supplementary figures and images for: Staphylococcal Bacterial Persister Cells, Biofilms, and Intracellular Infection Are Disrupted by JD1, a Membrane-Damaging Small Molecule
Source: mBio. 2021 Oct 12;12(5):e01801-21. doi: 10.1128/mBio.01801-21 (PMC8510524; doi:10.1128/mBio.01801-21)

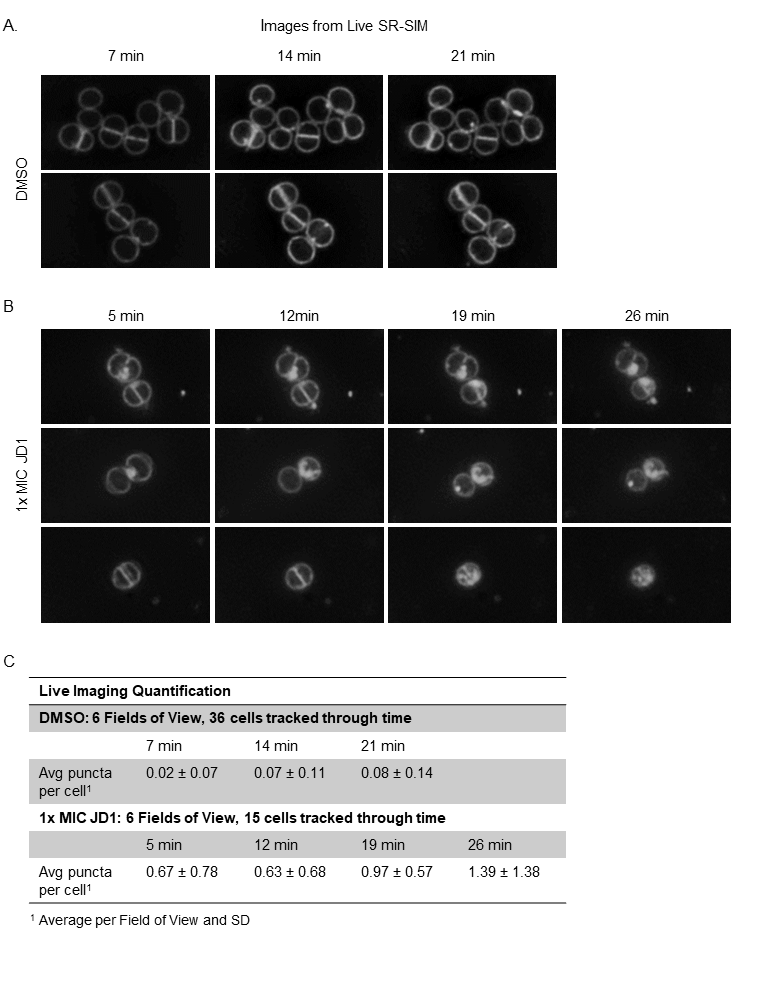

Supplement: FIG S1 [file mbio.01801-21-sf001.tif]

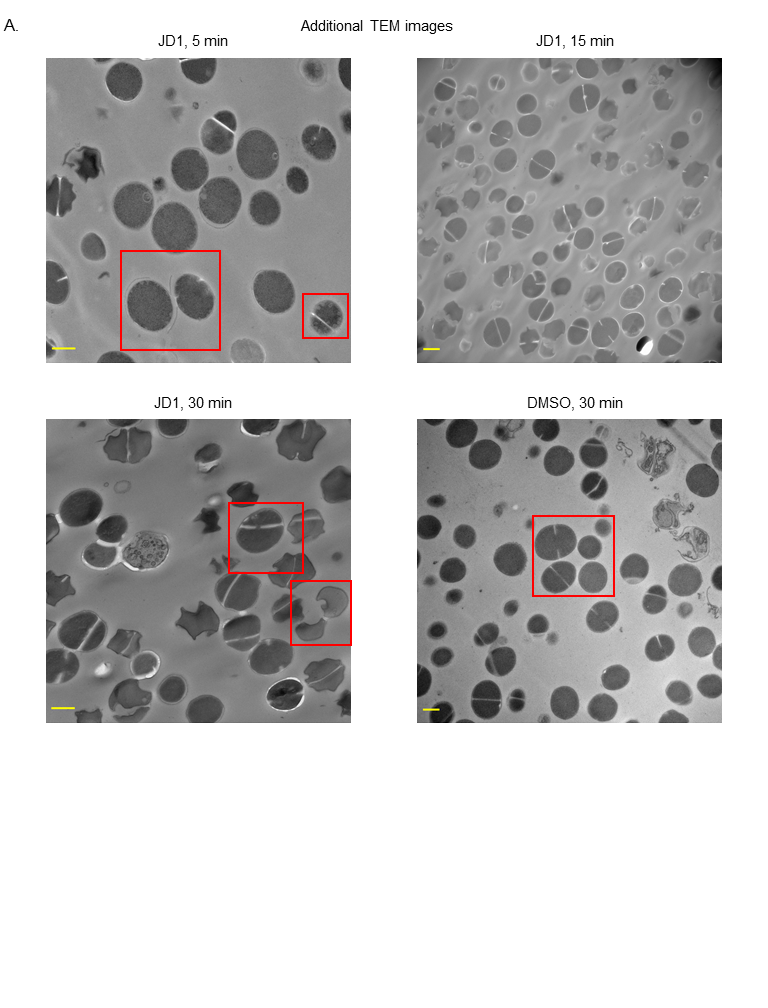

Supplement: FIG S2 [file mbio.01801-21-sf002.tif]

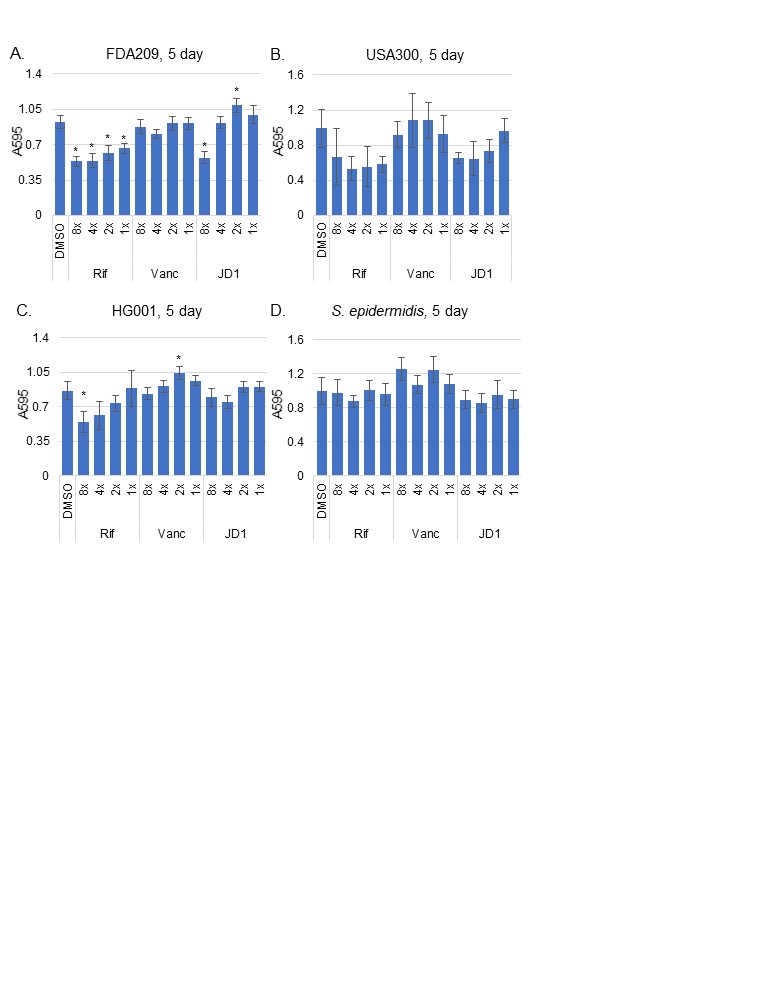

Supplement: FIG S3 [file mbio.01801-21-sf003.tif]

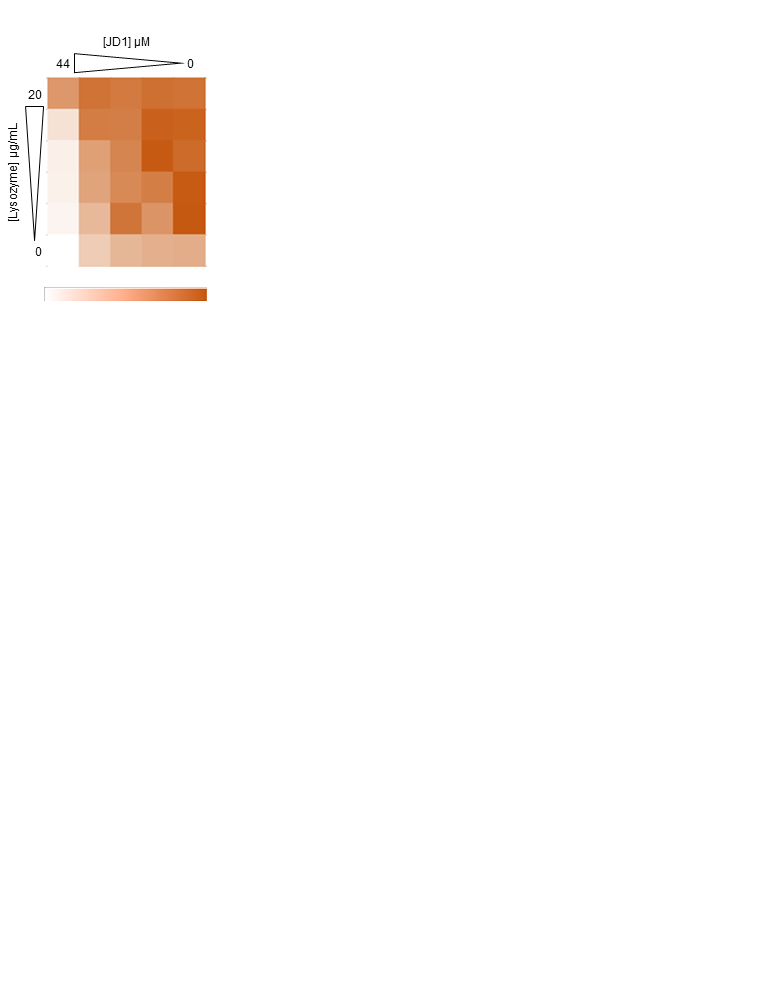

Supplement: FIG S4 [file mbio.01801-21-sf004.tif]
